# To Dope or Not to Dope: Neuroenhancement with Prescription Drugs and Drugs of Abuse among Swiss University Students

**DOI:** 10.1371/journal.pone.0077967

**Published:** 2013-11-13

**Authors:** Larissa J. Maier, Matthias E. Liechti, Fiona Herzig, Michael P. Schaub

**Affiliations:** 1 Swiss Research Institute for Public Health and Addiction, Associated Institute at the University of Zurich and WHO Collaborating Centre, Zurich, Switzerland; 2 Division of Clinical Pharmacology & Toxicology, Department of Biomedicine and Department of Internal Medicine, University Hospital Basel and University of Basel, Basel, Switzerland; 3 Division of Psychopathology and Clinical Intervention, University of Zurich, Zurich, Switzerland; California Pacific Medicial Center Research Institute, United States of America

## Abstract

**Background:**

Neuroenhancement is the use of substances by healthy subjects to enhance mood or cognitive function. The prevalence of neuroenhancement among Swiss university students is unknown. Investigating the prevalence of neuroenhancement among students is important to monitor problematic use and evaluate the necessity of prevention programs.

**Study aim:**

To describe the prevalence of the use of prescription medications and drugs of abuse for neuroenhancement among Swiss university students.

**Method:**

In this cross-sectional study, students at the University of Zurich, University of Basel, and Swiss Federal Institute of Technology Zurich were invited via e-mail to participate in an online survey.

**Results:**

A total of 28,118 students were contacted, and 6,275 students completed the survey. Across all of the institutions, 13.8% of the respondents indicated that they had used prescription drugs (7.6%) or drugs of abuse including alcohol (7.8%) at least once specifically for neuroenhancement. The most frequently used prescription drugs for neuroenhancement were methylphenidate (4.1%), sedatives (2.7%), and beta-blockers (1.2%). Alcohol was used for this purpose by 5.6% of the participants, followed by cannabis (2.5%), amphetamines (0.4%), and cocaine (0.2%). Arguments for neuroenhancement included increased learning (66.2%), relaxation or sleep improvement (51.2%), reduced nervousness (39.1%), coping with performance pressure (34.9%), increased performance (32.2%), and experimentation (20%). Neuroenhancement was significantly more prevalent among more senior students, students who reported higher levels of stress, and students who had previously used illicit drugs. Although “soft enhancers”, including coffee, energy drinks, vitamins, and tonics, were used daily in the month prior to an exam, prescription drugs or drugs of abuse were used much less frequently.

**Conclusions:**

A significant proportion of Swiss university students across most academic disciplines reported neuroenhancement with prescription drugs and drugs of abuse. However, these substances are rarely used on a daily basis and more sporadically used prior to exams.

## Introduction

In recent years, there has been growing public awareness of neuroenhancement, defined as the use of prescription drugs or other psychoactive substances by healthy individuals who try to improve their cognitive function or mood [1]–[4]. People may use potentially enhancing substances in situations of performance pressure if they are afraid that they will not fulfill others' or their own performance expectations [5]. Students appear to be at high-risk for using enhancing substances [2], [6]–[8]. Pharmacological interventions may affect the cognitive performance or mental state of a person [3]. The purpose of neuroenhancement is to improve cognitive function and emotional and motivational skills [9]. Several studies have reported that individuals seek neuroenhancement to enhance both cognitive function (e.g., alertness, attention, concentration, and memory) and psychological function (e.g., mood and sleep), which may indirectly enhance cognitive performance [10], [11]. Many studies in the field of neuroenhancement have only focused on the misuse of stimulant drugs, such as methylphenidate and amphetamines [2], [6], [12], [13], and have not investigated sedative drugs, which can have at least an indirect impact on cognitive performance. Prescription drugs, as well as alcohol and illicit drugs of abuse that are taken with the intent of improving mental performance, are subsumed under the term “neuroenhancers,” whereas non-prescription drugs, food supplements, and caffeine-containing products can be regarded as “soft enhancers” [14].

Some evidence suggests that neuroenhancement is widespread within academic institutions. A non-representative survey in *Nature* showed that 20% of all academics had taken methylphenidate, modafinil, or beta-blockers to improve cognitive performance [6], [7], [15]. Measures of the prevalence of neuroenhancement in the United States vary between 5% and 9% for high school students and between 5% and 35% for college students [12], [13], [16], [17]. The prevalence rates reported in these studies must be viewed critically because many studies have failed to ask participants about the reason why possible neuroenhancing substances are taken. Therefore, the recreational use of substances may have also contributed to these results [12]. The motivation for the non-medical use of prescription drugs or the use of alcohol and illicit drugs varies widely. The mean lifetime prevalence for “study drugs” in young adults in the United States is approximately 7%, and the past year prevalence of the illicit use of prescription stimulants is estimated to be 4% [18].

Several studies in Europe, the United States, and Australia have estimated the prevalence of neuroenhancement, although the findings differ greatly. In Europe, neuroenhancement does not appear to be as common as in the United States or Canada [2], [14], [19], [20]. Among German students, 1.55% used prescription stimulants, and 2.6% used illegal stimulants for neuroenhancement [2]. In the same study, male students and students with poor grades used more neuroenhancing substances than female students and students with good grades [2]. More recent studies, however, have reported a higher prevalence of neuroenhancement among students in Germany. A study of German university students reported a period prevalence rate of neuroenhancement of 7%, including the “time during studies” [21]. Another study found a 12-month prevalence of 20% for students when caffeine tablets were included as neuroenhancers [22]. A weakness of this study, however, was the definition of neuroenhancement and a questionable statistical approach because a 20% past year use in students appears very high.

Estimating how many students effectively practice neuroenhancement is difficult. Published data on neuroenhancement among Swiss students is lacking. According to a recent study, Swiss psychiatrists and general practitioners are faced with requests for neuroenhancers an average of once or twice per year, and nearly half of them (49.1%) reported that they decided on such requests pragmatically on a case-by-case basis [23].

Healthy individuals are usually unaware of the possible side effects of consuming prescription drugs for neuroenhancement. Even the known side effects of alcohol and illicit psychoactive substances can vary individually. Several studies of possible neuroenhancing substances showed that they exerted no or minimal effects on cognitive function. Positive effects on concentration, alertness, and attention were only documented for stimulants and modafinil [20], [24]–[26]. Additionally, no long-term studies of the negative effects of prescription drugs (e.g., investigations of tolerance and abuse potential in healthy individuals) have been performed [27]. However, Wulf (2009) introduced the theoretical view that neuroenhancement may disturb self-efficacy expectations because performance and outcomes become increasingly more attributed to the use of supposedly enhancing substances [28].

Students' and physicians' perceptions of neuroenhancement are rather negative. An Australian study showed that students were often skeptical about the potential benefits of stimulants with regard to the enhancement of cognitive function, and they were afraid of unknown side effects, mental health issues, and psychological dependence [29]. In a Canadian study, physicians did not feel comfortable prescribing cognition-enhancing substances for young adults, even if a hypothetical medication exists that is safe, effective, and without significant adverse side effects [30]. Students who are experienced with neuroenhancement did not perceive a difference (28% of respondents) or could not decide whether a difference exists (28%) between caffeinated substances and illicit or prescription stimulants, such as amphetamines or methylphenidate [31].

The relationship between experienced performance pressure and neuroenhancement has previously been investigated. In 2010, 34% of Swiss employees felt that they perceived chronic stress, whereas in the same survey in 2000, only 7% of the participants reported frequent high levels of stress [32]. The authors estimated that in the last 12 months, 6% of Swiss employees had taken substances for cognitive enhancement, and 15% had consumed substances to relax and switch off after stressful days at work [32]. In a German study, 31% of students felt strong performance pressure and were afraid of being unable to cope with demands concerning their studies [14].

Which substances are used for neuroenhancement is poorly understood. Medications to treat attention deficit disorder (ADD) and attention-deficit/hyperactivity disorder (ADHD), such as methylphenidate and the wake-promoting agent modafinil, are the pharmaceuticals that are generally discussed in the bioethical debate on neuroenhancement [7], [24], [33]. ADHD is the most prevalent neuropsychiatric disorder in childhood [15]. In Switzerland, the number of people who obtain ADHD medications increased by 42% from 2006 to 2009, and the quantity of methylphenidate used per person and per year increased from 5,600 mg to 6,200 mg, respectively [34]. A history of multiple substance use is a risk-factor for the non-medical use of methylphenidate [35]. The co-occurrence of illicit drug use and the non-medical use of prescription stimulants and the occurrence of ADD/ADHD patients who misuse their medication have also been reported among college students [8], [36]. Based on a study of United States college students, Novak and colleagues estimated that one-quarter of ADD/ADHD patients consume their medications in a manner that differs from the intended prescription of the medication [36]. Different medications are used to treat ADD/ADHD in different countries. In the United States, dextroamphetamine and mixed amphetamine salts are used to treat ADD/ADHD, whereas these medications are not licensed in Switzerland or Germany [37], [38]. As a result, methylphenidate is the most commonly prescribed ADD/ADHD medication in Switzerland [34].

To overcome the lack of empirical data on neuroenhancement among Swiss university students, we created a cross-sectional online survey to investigate whether the prevalence of use was similar to the use in other countries and explore the relevant factors, such as study-related stress, possible arguments for neuroenhancement, sources of supply, and preliminary attitudes about neuroenhancement. The students were asked to report their non-medical use of prescription drugs and drugs of abuse and the purpose of consuming each substance. In contrast to earlier studies, we also inquired about both the recreational and neuroenhancement uses of alcohol. The aim of the present study was to identify several substances that are used by students to improve cognitive performance and mood and describe the extent of prescription drug, alcohol, and illicit psychoactive substance use for the purpose of neuroenhancement among Swiss university students. Investigating the prevalence of neuroenhancement among student populations is important to monitor problematic use and evaluate the necessity of prevention programs.

## Methods

### Study procedures and sample

This social-empirical cross-sectional study was performed from December 2012 to January 2013. A total of 28,118 students from three different educational institutions in Switzerland were contacted by e-mail and asked to complete an online questionnaire about the use of psychoactive substances. The study was approved by the ethics committee of the Philosophical Faculty of the University of Zurich and Ethics Committee of Basel. The informational message distributed to potential subjects invited them to participate, explained the rationale for the study, and stated that the study was reviewed by the ethics committee of the Philosophical Faculty of the University of Zurich and the Ethics Committee of Basel, who declared no objection. The message to the participants also provided a web link that provided more information about the study: the study was absolutely voluntary, personal data would be anonymized and stored on a secure server, and the participants had the right to withdraw from the study at any time without any consequences except their exclusion from the drawing for a tablet computer. The participants were also allowed to ask the study coordinator questions. Informed consent was recorded when the participants left the study information web page and began the survey by clicking on the survey start button.

The survey was conducted among students at the University of Zurich (UZH), University of Basel (UniBas), and Swiss Federal Institute of Technology Zurich (ETHZ). The contacts established through the mailing lists of each university included 5,000 students from UZH, 12,337 students from ETHZ, and 10,781 students from UniBas. The students were told that the aim of the study was to determine whether the use of neuroenhancing substances was common at Swiss universities. The term “brain doping” was defined as the intake of any substances to enhance cognitive function. The participants were also informed that they could win a tablet computer by providing their e-mail address, which was stored separately from the survey data. The final sample size was 6,275 students.

### Measures

The questionnaire was specifically designed for the present study at the Swiss Research Institute for Public Health and Addiction. Some questions were taken from existing questionnaires on neuroenhancement (e.g., Middendorf et al. [14]), and questions concerning the consumption patterns and consumption frequency of different substances were based on the recommendations of Swiss Addiction Monitoring [39]. At the beginning of the questionnaire, neuroenhancement was defined as the use of prescription drugs or other psychoactive substances (e.g., drugs of abuse, such as cannabis) to directly or indirectly enhance brain function (e.g., concentration, alertness, and a reduction of nervousness). The questionnaire included additional questions on the use of coffee, caffeine tablets, and herbal products. The participants were informed that completion of the questionnaire would take approximately 15 min. The participants were first asked to indicate their university, major, semester of study, age, gender, and whether they studied full-time or part-time. The participants were also asked to rate the degree of stress and performance pressure they felt with regard to education, work, leisure, and family on a scale from 1 to 5. The subjects were then asked whether they had heard that prescription drugs, drugs of abuse, and other substances are used to enhance cognitive function. The subjects were also asked whether they had ever used one or more of the substances that had been listed without having a clear medical indication for doing so (i.e., lifetime prevalence of non-medical use for recreational or neuroenhancement purposes). If the students responded that they had used a substance without a clear medical indication, then they had to indicate the purpose of use (e.g., recreationally at parties, cognitive enhancement for studying, in stressful situations, during exams, to enhance performance, or to learn more efficiently or for longer periods of time). For each substance, the participants also had to indicate the frequency of use within the last 30 days prior to the last exam and whether their expectations regarding the medication's effects were met. These questions were asked for the following groups of substances: methylphenidate, modafinil, activating antidepressants including selective serotonin reuptake inhibitors (SSRIs), anti-dementia agents, sedatives/hypnotics, herbal sedatives (e.g., St. John's Wort, common valerian), beta-blockers, vitamins and tonics (e.g., *Gingko biloba*, zinc, vitamin pills), alcohol, cannabis, cocaine, amphetamines (amphetamine, methamphetamine), ecstasy (methylenedioxymethamphetamine), γ-hydroxybutyrate/γ-butyrolactone (GHB/GBL; liquid ecstasy), coffee, caffeine tablets, and energy drinks. A series of questions asked about problems that occurred as a result of the consumption of substances for the purpose of neuroenhancement. The participants were asked whether they experienced problems with education, family, friends, police, finances, or health. Health-related questions asked whether the subjects had experienced the following symptoms: passing out, anxiety and panic attacks, aggressiveness, depressive symptoms, loss of appetite, increased sweating, sleeping disorders, heart palpitations, tachycardia, headache, nervousness, or accidents. The students were asked to indicate the source from which they obtained the prescription drugs (e.g., Internet, physician, pediatrician, pharmacy, person with a prescription, dealer, parents, and colleagues). According to a survey among German university students [14], the subjects were then asked more specifically about their motives for brain doping (e.g., to learn more quickly and more efficiently, to reduce nervousness and performance anxiety, to increase performance, to relax and improve sleep, to reduce high performance pressure, to cope with competitive pressure, curiosity and experimentation, and because others use it) and whether they generally used these substances for exam preparation, during exams, or in stressful life situations. This question was asked for all of the substances, including soft enhancers such as coffee or energy drinks.

The students were asked to report how many people they knew were using prescription drugs or psychoactive substances for studying. Additionally, the subjects were asked whether they had ever been diagnosed with a psychiatric disorder, including ADHD, depression, anxiety, and other disorders, and whether they were regularly taking any medications. The last question was about personal justifiable motives for the consumption of enhancing substances. The students could choose one or more options that they believed justified the use of neuroenhancers (e.g., suppress fatigue, manage sleeping disorders, manage time, manage conflicts, address the pressure of competition, manage nervousness, manage poor mood, alleviate pain, enhance charisma, treat depression, treat influenza, and medical advice), or they could say that neuroenhancement was unacceptable in any situation.

### Definitions and statistical analyses

The analyses were performed using SPSS 20 for Windows. Descriptive statistics were used to evaluate the questions. Cross tables and Pearson's *χ^2^* tests were used to test for significant group differences in the prevalence of use. Three different categories were used. The category “prescription drugs” included methylphenidate, modafinil, antidepressants, anti-dementia agents, sedatives, and beta-blockers. The category “drugs of abuse including alcohol” included all substances of abuse, including alcohol, cannabis, cocaine, amphetamines, ecstasy, and GHB/GBL. The term “neuroenhancement” is used here for the reported use of both prescription drugs and drugs of abuse, including alcohol, if the students reported that they had used any of these substances to enhance cognitive function. A third category called “soft enhancers” [14] included herbal sedatives, vitamins and tonics, coffee, caffeine tablets, and energy drinks. These substances were also taken by the students with the purpose of improving mental performance, although they were available in drug stores without a prescription. Soft enhancers were not included in the neuroenhancement drug category.

## Results

### Sample description

Response rates and participant characteristics are shown in Table 1. The participants were 17 to 68 years old, with an average age of 23.18 years. The number of semesters completed ranged from one to 33, with an average of five semesters. Gender was equally distributed among the entire sample. Most of the respondents from UZH and UniBas were women, whereas the majority of the ETHZ students were men. Most of the participants had a full-time study workload. The students from ETHZ were primarily full-time students and were less frequently employed during their studies. Students in psychology, law, economics, and sports were frequently working in addition to studying.

**Table 1:** Response rates and participant characteristics (*N* = 6275).

	Institution			
	UZH^1^ (*n* = 404)	ETHZ^2^ (*n* = 3347)	UniBas^3^ (*n* = 2524)	Total (*N* = 6275)
Response rate	8.1% (404 of 5000)	27.1% (3347 of 12337)	23.4% (2524 of 10781)	22.3% (6275 of 28118)
Gender				
Male	25.5% (103)	61.4% (2055)	41% (1034)	50.9% (3192)
Female	74.5% (301)	38.6% (1292)	59% (1490)	49.1% (3083)
Mean age (years)	24.85 (*SD* = 5.42)	22.37 (*SD* = 2.65)	23.98 (*SD* = 5.01)	23.18 (*SD* = 4.06)
Number of semesters	6.19 (*SD* = 4.01)	4.86 (*SD* = 2.99)	5.5 (*SD* = 3.47)	5.21 (*SD* = 3.29)
Study workload				
Full-time	84.7% (342)	98.2% (3287)	87.2% (2200)	92.9% (5829)
Part-time	15.3% (62)	1.8% (60)	12.8% (324)	7.1% (446)
Employment during studies				
Yes	71.3% (288)	31.6% (1058)	59% (1488)	45.2% (2834)
No	28.7% (116)	68.4% (2289)	41% (1036)	54.8% (3241)

1 University of Zurich.

2 Swiss Federal Institute of Technology Zurich.

3 University of Basel.

### Prevalence rates of neuroenhancement

The majority of the respondents (93.7%) reported that they had heard that prescription drugs, drugs of abuse, and other substances could be used to enhance cognitive function. Of the study population, 868 subjects (13.8%) had already used prescription drugs or drugs of abuse, including alcohol, at least once with the purpose of enhancing cognitive function (Table 2). Prescription drugs had been used in 7.6% of the sample, and drugs of abuse, including alcohol, had been used by 7.8% (Table 2). Only 1.5% of the respondents reported using both prescription drugs and drugs of abuse for neuroenhancement purposes. As shown in Table 3, the most frequently used prescription drugs for academic performance enhancement were methylphenidate and sedatives. Alcohol and cannabis were the most frequently used drugs of abuse, whereas stimulant drugs, such as amphetamines, cocaine, and ecstasy, were rarely used for the purpose of neuroenhancement. Among the soft enhancers, half of the students used coffee, and one-third used energy drinks explicitly to enhance their cognitive function. The use of caffeine tablets for this purpose was not very prevalent. The daily use of substances for neuroenhancement in the month prior to an exam was only reported for soft enhancers, such as coffee, vitamins and tonics, and energy drinks, but not for prescription drugs or drugs of abuse (Table 3). One-third of the students (33%) reported daily use of at least one soft enhancer, whereas only 1.8% reported the daily use of a prescription drug or drug of abuse, including alcohol, for the purpose of neuroenhancement. The majority of the students who took any substance for neuroenhancement reported that such substances met their expectations with regard to the anticipated effects, with the exception of anti-dementia agents, for which most of the students were dissatisfied (Table 3). More than half of the respondents (52.7%) used neuroenhancement or soft enhancers for exam preparation, and 24.3% used any of these substances during the exam itself. Among the students, 19.8% also noted that they used neuroenhancement or soft enhancers during stressful life situations in general. Students who used neuroenhancers (*n* = 868) more frequently reported the use of enhancing substances, including soft enhancers, during exam preparation (69.9%), exams (37.7%), or stressful life situations in general (36.2%) compared with students who never used neuroenhancers and only used vitamins and tonics, caffeine-containing products, or herbal products to cope with stressful situations.

**Table 2:** Lifetime prevalence of neuroenhancement according to substance categories and study site (*N* = 6275).

	Institution			
	UZH^1^ (*n* = 404)	ETHZ^2^ (*n* = 3347)	UniBas^3^ (*n* = 2524)	Total (*N* = 6275)
Prescription drugs				
Men	12.6% (13)	6.6% (135)	8.3% (86)	7.3% (234)
Women	12% (36)	6% (78)	8.5% (127)	7.8% (241)
Total	12.1% (49)	6.4% (213)	8.4% (213)	7.6% (475)
Drugs of abuse including alcohol				
Men	11.7% (12)	8.5% (174)*	9.5% (98)**	8.9% (284)***
Women	8.3% (25)	6.3% (81)	6.6% (98)	6.6% (204)
Total	9.2% (37)	7.6% (255)	7.8% (196)	7.8% (488)
Prescription drugs AND drugs of abuse including alcohol				
Men	1.9% (2)	1.6% (33)	1.8% (19)	1.7% (54)
Women	2.3% (7)	1% (13)	1.4% (21)	1.3% (41)
Total	2.2% (9)	1.4% (46)	1.6% (40)	1.5% (95)
Prescription drugs OR drugs of abuse including alcohol				
Men	22.3% (23)	13.4% (276)*	16% (165)	14.5% (464)
Women	17.9% (54)	11.3% (145)	13.7% (204)	13.1% (404)
Total	19.1% (77)	12.6% (422)	14.6% (369)	13.8% (868)

* *p*<0.05,

** *p*<0.01,

*** *p*<0.001, compared with women.

1 University of Zurich.

2 Swiss Federal Institute of Technology Zurich.

3 University of Basel.

**Table 3:** Prevalence of neuroenhancement and consumption patterns of substances used for neuroenhancement (*N* = 6275).

	All types of use	Recreational use	Use for neuroenhancement			
	Lifetime prevalence	Lifetime prevalence	Lifetime prevalence	Last month prior to exam	Daily use prior to exam	Expectations fulfilled^1^
Prescription drugs						
Methylphenidate	5.8% (367)	2.3% (145)	4.1% (255)	2.6% (163)	0.4% (22)	67.5% (172)
Modafinil	0.4% (25)	0.1% (5)	0.3% (22)	0.2% (15)	0.04% (3)	68.2% (15)
Antidepressants	1.6% (97)	0.3% (17)	0.5% (32)	0.4% (26)	0.2% (14)	59.4% (19)
Anti-dementia agents	0.1% (8)	—	0.1% (8)	0.1% (7)	0.03% (2)	37.5% (3)
Sedatives	5.8% (364)	3.4% (215)	2.7% (170)	2.1% (133)	0.2% (14)	75.9 (129)
Beta-blockers	1.7% (108)	0.5% (30)	1.2% (74)	0.7% (45)	0.1% (6)	70.3% (52)
Drugs of abuse including alcohol						
Alcohol	93.4% (5688)	90.2% (5660)	5.6% (350)	5.1% (320)	0.4% (22)	87.1% (305)
Cannabis	45.1% (2741)	43.3% (2720)	2.5% (158)	1.8% (115)	0.6% (37)	93% (147)
Cocaine	4.3% (264)	4.2% (262)	0.2% (12)	0.1% (7)	0.02% (1)	75% (9)
Amphetamines	3.9% (239)	3.7% (231)	0.4% (26)	0.3% (17)	0.03% (2)	84.6% (22)
Ecstasy	5.6% (337)	5.2% (327)	0.1% (4)	0.02% (1)	—	100% (4)
GHB/GBL	0.9% (56)	0.9% (54)	—	—	—	—
“Soft enhancers” (non-prescription drugs, food supplements, caffeine-containing products, etc.)						
Herbal sedatives^2^	29.1% (1804)	12.6% (793)	18.2% (1143)	13.2% (830)	1.4% (86)	63.9% (730)
Vitamins and tonics^3^	40.6% (2505)	25.1% (1575)	18.2% (1140)	14.9% (933)	5.2% (328)	67.3% (766)
Coffee	86.3% (5212)	75.4% (4735)	53.2% (3340)	49.1% (3081)	28.3% (1776)	84.9% (2834)
Caffeine tablets	7.4% (444)	3.2% (200)	4.4% (276)	2.6% (166)	0.3% (18)	69.2% (191)
Energy drinks	67.5% (4069)	53.8% (3375)	35.9% (2253)	29.7% (1862)	4% (250)	82.1% (1849)

1 Percent of students who used neuroenhancement.

2 St. John's wort, common valerian.

3 *Gingko biloba*, zinc, vitamin tablets.

In our sample, 5.8% of the students (6.9% of men and 4.7% of women) reported the non-medical use of methylphenidate for either neuroenhancement or recreational purposes. The lifetime prevalence rates of methylphenidate use for neuroenhancement were 4.6% for men and 3.5% for women. The lifetime prevalence of recreational methylphenidate use was significantly higher among men (3.1%) than among women (1.5%; *p* = 0.007). The nasal administration of methylphenidate was more common among recreational users (13.4%) compared with students who used it for neuroenhancement purposes (6%). As shown in Table 2, male students used drugs of abuse for neuroenhancement purposes significantly more often than female students. Female students used soft enhancers more often for enhancing purposes (herbal sedatives, *p*<0.001; vitamins and tonics, *p*<0.001; coffee, *p* = 0.001; caffeine tablets, *p* = 0.001; energy drinks, *p*<0.001), whereas male students were more experienced with the recreational use of methylphenidate (*p* = 0.007), vitamins and tonics (*p* = 0.005), caffeine tablets (*p*<0.001), and energy drinks (*p*<0.001).

The prevalence rate of neuroenhancement varied slightly across different study majors. Almost one in five architecture students had used neuroenhancers to improve cognitive function or mood (Table 4). A high lifetime prevalence of neuroenhancement was also found in journalism and communication, chemistry, and economics students. Mathematics and sports students were less experienced with neuroenhancement (Table 4).

**Table 4:** Lifetime prevalence of neuroenhancement and experiences with illicit drugs of abuse by academic discipline (*N* = 6275).

	Neuroenhancement	Lifetime experience with illicit drugs of abuse^2^	
Major discipline^1^	Prescription drugs OR drugs of abuse including alcohol	Including cannabis	Excluding cannabis
Biology (*n* = 385)	14.5% (56)	44.2% (170)	5.6% (21)
Chemistry (*n* = 205)	17.6% (36)	43.4% (89)	9.8% (19)
Medicine (*n* = 395)	16.2% (64)	46.6% (184)	7.3% (28)
Psychology (*n* = 339)	14.5% (49)	51.9% (176)	9.9% (32)
Journalism and communication (*n* = 66)	18.2% (12)	60.6% (40)	14.1% (9)
Law (*n* = 249)	14.5% (36)	43.4% (108)	9% (22)
Economics (*n* = 321)	17.1% (55)	49.2% (158)	9.7% (30)
Architecture (*n* = 321)	19.6% (63)	56.4% (181)	14.1% (43)
Sports (*n* = 229)	7% (16)	34.1% (78)	5.8% (13)
Mechanical engineering (*n* = 584)	10.6% (62)	38.2% (223)	6.8% (38)
Mathematics (*n* = 174)	8.6% (15)	36.2% (63)	6.6% (11)
Pharmaceutical sciences (*n* = 341)	16.1% (55)	37.5% (128)	6.1% (20)
Physics (*n* = 209)	11.5% (24)	37.3% (78)	3% (6)
Environmental sciences (*n* = 219)	11.4% (25)	45.2% (99)	10.9% (23)
Total (*N* = 6275)	13.8% (868)	44.3% (2777)	7.8% (491)

1 Other (*n* = 2226): politics, veterinary medicine, theology, philosophy, history, literature, linguistics, art history, dental medicine, information technology, geography etc.

2 Cocaine, amphetamines, ecstasy, GHB/GBL, with/without cannabis.

### Neuroenhancement and experiences with illicit drugs of abuse

The lifetime prevalence of illicit drug use for any purpose was 44.3% when including cannabis and 7.8% when excluding cannabis (Table 4). Across academic disciplines, the prevalence rate of illicit drug use ranged from 34.1% to 60.6% when cannabis was included and from 3% to 14.1% when cannabis was excluded (see Table 4 for academic discipline results). The primary purpose of using illicit drugs was recreational rather than for neuroenhancement purposes (Table 3). Students who had experience with illicit drugs of abuse used prescription drugs or drugs of abuse for neuroenhancement significantly more often than students who had never tried illicit substances (Table 5). These findings were consistent across all of the study sites.

**Table 5:** Experience with neuroenhancement and illicit drugs of abuse (*N* = 6275).

	Neuroenhancement	
	Lifetime experience with illicit drugs of abuse (*n* = 2777)	Never tried illicit drugs of abuse (*n* = 3498)
Prescription drugs	10.8% (299)***	5% (176)
Drugs of abuse including alcohol	13.1% (365)***	3.5% (123)
Prescription drugs AND drugs of abuse including alcohol	2.9% (81)***	0.4% (14)
Prescription drugs OR drugs of abuse including alcohol	21% (583)***	8.1% (285)

* *p*<0.05,

** *p*<0.01,

*** *p*<0.001, compared with people who never tried illicit drugs of abuse.

One-third of the students who used neuroenhancers (33.9%) knew one or two people who already used prescription drugs to improve their cognitive function. Only 40.1% of the students who experienced neuroenhancement did not know anyone who took prescription drugs for neuroenhancement purposes. Of the students who never used neuroenhancement, 64.5% reported not knowing anyone who consumed prescription drugs to enhance academic performance. Half of the students who used neuroenhancement (49.3%) knew at least one person who had tried to enhance cognitive function by using drugs of abuse, whereas only 29.3% of the non-users knew people who had done so. Of the students who used neuroenhancement, 70.9% knew eight or more people who had used soft enhancers, such as coffee or energy drinks. Additionally, 57.1% of the students without neuroenhancement knew at least eight people who had used soft enhancers to improve cognitive performance.

### Perceived stress and neuroenhancement

Students who reported higher levels of performance pressure with regard to education, work, leisure, or family were also more experienced with neuroenhancement. Of the respondents who rated their stress highest for work, 28% had consumed prescription drugs or drugs of abuse for neuroenhancement purposes. Of the students with high subjective performance pressure in the area of education, 18.2% reported neuroenhancement, although 14.6% of the students who felt no performance pressure at all also practiced neuroenhancement. Students who reported experiencing lower education- or work-related performance pressure reported a higher lifetime use of illicit drugs of abuse. The most commonly experienced side effects after consuming neuroenhancers were nervousness (27.1%), sleeping disorders (26.4%), and headaches (25%). The participants also reported experiencing depressive states (18.1%), loss of appetite (17.9%), and tachycardia (15.8%). Problems with family (6.3%) or friends (3.8%) caused by neuroenhancement were uncommon. Some students had experienced an anxiety attack (7.4%) or felt aggressive (6.6%) while under the influence of neuroenhancers, and some students reported problems with their education (5.1%) while using neuroenhancers. Only a few students had financial problems (2.4%), problems with the police (2.3%), accidents (0.9%), or passed out (2.5%) because of the consumption of neuroenhancers. Many students did not report problems related to neuroenhancement (38.1%).

### Source of supply

Of the participants who used prescription drugs or drugs of abuse for neuroenhancement purposes, 15.4% received their prescription drugs for neuroenhancement from a doctor. Other students (14.7%) and people with a prescription (12.9%) were another common source of neuroenhancement prescription drugs. Of the respondents, 10.5% purchased their prescription drugs at a pharmacy, and only 4.1% of the students purchased prescription drugs or illicit drugs of abuse for neuroenhancement on the Internet. One individual stated that he had received prescription drugs from a pediatrician, and 5.9% of the respondents reported that their parents were the source of the prescription drugs or illicit drugs of abuse for neuroenhancement. The respondents reported that they obtained prescription drugs to enhance cognitive function from psychiatrists (6.3%) and also obtained prescription drugs and illicit drugs of abuse for neuroenhancement from dealers (8.1%).

### Subjects with ADHD diagnosis

Forty-one of 109 (37.6%) students who had ever been diagnosed with ADD/ADHD were taking medications regularly. Thirty-six (33%) students with an ADD/ADHD diagnosis indicated that they had misused methylphenidate. Ten students (9.2%) reported the recreational use of methylphenidate, and 29 students reported methylphenidate use for cognitive enhancement (26.6%).

When excluding subjects with an ADHD diagnosis, the prevalence for methylphenidate use was 5.4% compared with 5.8% in the entire sample. The prevalence rates of recreational or neuroenhancement use of methylphenidate were 2.2% and 3.7%, respectively, when excluding subjects with ADHD compared with 2.3% and 4.1%, respectively, in the entire sample.

### Arguments for/against neuroenhancement

Arguments for the use of neuroenhancers included increased learning (66.2%), relaxation or sleep improvement (51.2%), reduced nervousness (39.1%), coping with performance pressure (34.9%), increased performance (32.2%), and experimentation (20%). Of the students who were experienced with neuroenhancement, only 9.8% mentioned competitive pressure and 2.8% mentioned other people's use of substances as motives for the use of enhancing substances.

Many students would accept the consumption of prescription drugs and drugs of abuse to enhance cognitive performance if the use of these substances was based on professional medical advice (44.5%) or the treatment of a disease (42.8%), a diagnosed psychological disorder (38%), or a sleeping disorder (36.6%). Motives such as enhancing cognition, alertness, and memory (40%), reducing pain (37%), suppressing fatigue (43%), or reducing nervousness (30.8%) would also justify neuroenhancement. The use of substances solely to enhance charisma (1.5%) or mood (5.4%), to solve conflicts (2.7%), or to manage the pressures of competition (6.7%) and limited time (14.6%) was acceptable only among a few students. Only 15.5% of the students reported that they see no reason for neuroenhancement at all.

## Discussion

The main finding of the present survey was that a significant proportion of Swiss university students reported a lifetime use of prescription drugs or drugs of abuse to enhance academic performance. Additionally, this finding was consistent across three different institutions and across study majors. Of the 6,275 respondents, 868 (13.8%) had experience with neuroenhancement. The most commonly used substances were methylphenidate (4.1%), sedatives (2.7%), alcohol (5.6%), and cannabis (2.5%). Although neuroenhancement was used especially for exam preparation (69.9%), the daily use of prescription drugs or drugs of abuse as neuroenhancers was uncommon, even prior to an exam.

The high lifetime prevalence rate of neuroenhancement found in the present study depends on the definition of “neuroenhancement,” which in our case also included drugs with sedative effects. Clearly, sedatives are not used to directly enhance learning processes, but they can influence academic performance in a desirable way. A well-rested brain likely learns more efficiently. Thus, for example, the consumption of cannabis or alcohol with the purpose of calming oneself, turning off reoccurring thoughts, and falling asleep would allow one to be more vigilant and increase concentration the following morning. Thus, the use of alcohol and cannabis in such a case could also be considered a form of neuroenhancement. Only a few previous studies have included sedative substances in their concept of neuroenhancement [14], [19]. According to the definitions provided in earlier studies, we performed a supplemental analysis in which we excluded all sedatives, including alcohol and cannabis, and only included methylphenidate, modafinil, antidepressants, anti-dementia agents, beta-blockers, cocaine, and amphetamines in the neuroenhancement category. This analysis resulted in a considerably lower overall prevalence of neuroenhancement (5.9%) in our complete sample (Table 6).

**Table 6:** Lifetime prevalence of neuroenhancement according to substance categories and study site based on directly cognitive-enhancing neuroenhancement substances, including methylphenidate, modafinil, antidepressants, anti-dementia agents, beta-blockers, cocaine, and amphetamines (*N* = 6275).

		Institution		
	UZH (*n* = 404)	ETHZ (*n* = 3347)	UniBas (*n* = 2524)	Total (*N* = 6275)
Prescription drugs				
Men	10.7% (11)	5.1% (104)*	7.2% (74)	5.9% (189)
Women	7.3% (22)	3.7% (48)	6.6% (98)	5.4% (168)
Total	8.2% (33)	4.5% (152)	6.8% (172)	5.7% (357)
Drugs of abuse				
Men	1% (1)	0.7% (15)	0.7% (7)	0.7% (23)
Women	0.3% (1)	0.3% (4)	0.5% (7)	0.4% (12)
Total	0.5% (2)	0.6% (19)	0.6% (14)	0.6% (35)
Prescription drugs AND drugs of abuse				
Men	-	0.4% (9)	0.2% (2)	0.3% (11)
Women	0.3% (1)	0.3% (4)	0.2% (3)	0.3% (8)
Total	0.2% (1)	0.4% (13)	0.2% (5)	0.3% (19)
Prescription drugs OR drugs of abuse				
Men	11.7% (12)	5.4% (110)*	7.6% (79)	6.3% (201)
Women	7.3% (22)	3.7% (48)	6.8% (102)	5.6% (172)
Total	8.4% (34)	4.7% (158)	7.2% (182)	5.9% (373)

* *p*<0.05,

** *p*<0.01,

*** *p*<0.001, compared with women.

The primary motivation of users was to learn more quickly and more efficiently, which could be expected to result in enhanced academic performance. Soft enhancers, including coffee, energy drinks, vitamins and tonics, and herbal sedatives, are common among students in daily life, and the use of these substances also seems to be socially accepted, whereas neuroenhancement was only considered justified in certain situations where people were disadvantaged.

Methylphenidate was the most prevalent prescription drug used for neuroenhancement in the present study. The numbers of people who obtain ADD/ADHD medications and the amount of medication in Switzerland have increased over recent years [34]. The present study included both healthy individuals and people with a diagnosis of ADD/ADHD who misused their medication who consumed prescription drugs and drugs of abuse for neuroenhancement purposes. Misuse in patients is defined as the intake of higher doses of the medications [8] than prescribed or using another mode of application (intranasal or intravenous) than the one prescribed by a doctor [36]. In the present study, the recreational use of methylphenidate was more commonly associated with nasal administration (13.4%) compared with its use for neuroenhancement purposes (6%). Previous surveys often excluded subjects with an ADD/ADHD diagnosis to prevent biasing outcomes with regard to the prevalence of prescription drug use [12], [20], [40]. In the present study, only 41 students (37.6%) regularly took ADD/ADHD medications among 109 students who had ever been diagnosed with ADD/ADHD. Of the students who were ever diagnosed with ADD/ADHD, 36 (33%) had misused methylphenidate. Notably, only 10 students reported recreational use (9.2% of these students had a previous diagnosis of ADHD), whereas 29 students reported using the medication for cognitive enhancement (26.6%). The findings of the present study are consistent with previous research on ADD/ADHD patients and the misuse of their medications [36], [41]. The prevalence of use of methylphenidate for neuroenhancement purposes only slightly changed when subjects with an ADHD diagnosis were excluded. Therefore, the results were not confounded by ADHD diagnosis.

Female students used soft enhancers for neuroenhancement significantly more often than male students. This finding is consistent with a study conducted at German universities, in which the proportion of female students who used soft enhancers to enhance cognitive function was twice as large as in male students [14]. Concerning the use of drugs for recreational purposes, male students had significantly higher prevalence rates of methylphenidate, caffeine tablet, energy drink, and vitamin and tonic use. A possible explanation for these results might be that female students expect greater effects on cognitive performance and well-being from soft enhancers, whereas men prefer using prescription or illicit stimulants to improve their academic performance.

Students with experiences of illegal psychoactive substances had generally more commonly used prescription drugs or drugs of abuse (or both) to enhance cognitive function. This finding is consistent with previous research on the non-medical use of prescription stimulants [8], [35]. We also confirmed that students who used neuroenhancement showed more risky health behaviors compared with other students [14], [36].

Unlike studies in Germany, we did not find a higher prevalence of neuroenhancement in sports students or students in the pharmaceutical sciences or medicine compared with other study majors [14], [22]. The underlying explanation for this result should be further investigated in future research. Neuroenhancement was reported by fewer ETHZ students compared with the other two universities, although the highest prevalence rate was found among students who majored in architecture (ETHZ). A possible explanation for this observation might be that architecture students typically have to complete projects in a short period of time. Previous studies may have failed to detect the high prevalence of neuroenhancement among architecture students because they combined all of the technical sciences [14] or did not categorize the students by study major [2], [19], [22].

More senior students generally had more experience with neuroenhancement. These findings are consistent with the findings from German higher education institutions [14]. Only one German study found a higher prevalence of neuroenhancement among students in the first semester compared with senior students [22]. Students at UZH had a higher mean age compared with the other institutions. They had already been enrolled in education for a longer period of time, they were more likely to have been employed during their studies, and they had more experience with neuroenhancement, in contrast to students from other institutions. The higher prevalence of neuroenhancement among UZH students likely reflects their older age, longer duration of education, and additional job pressures as opposed to some particular characteristic of this institution. Additionally, the number of students from UZH was relatively small, and recruiting at that institution was not identical to the other institutions, which may have resulted in an additional selection bias.

Students who felt high performance pressure were more likely to consume prescription drugs or psychoactive substances (or both) to enhance cognitive function. High performance pressure at work led to a higher prevalence of neuroenhancement (28%). Compared with other students, students from UZH were more likely to be employed and felt higher performance pressure at work and in education simultaneously; therefore, this pressure could explain the higher prevalence rates of neuroenhancement at UZH. Consistent with these results, Australian university students believed that the use of prescription and illicit stimulants was related to attempts to manage both social life and educational requirements [29]. The aim of neuroenhancement may be not only to improve academic performance but also to enhance work-life balance.

A negative relationship was found between illicit substance use and performance pressure. Students who reported low performance pressure in the areas of education and work reported more illicit drug use. Therefore, students with high performance pressure may not allow themselves to consume illicit drugs.

Our findings in this report have several limitations. First, the study did not include information from all of the students at each of the three Swiss institutions because not every student had agreed to receive study invitation e-mails. This limitation was particularly problematic for UZH but not for UniBas, where all of the students were invited. Second, response rates from online surveys are typically quite low and subject to selection bias. However, response rates in the present study were as high as 23.4% and 27.1% at UniBas and ETHZ, respectively, but within the expected low range at UZH (8.1%). Third, all of the participants were from the German-speaking part of Switzerland (Zurich and Basel), and the results may not be generalizable to other Swiss universities, particularly universities in the French-speaking part of Switzerland. However, we recruited a very heterogeneous sample with respect to all major academic disciplines, in contrast to earlier studies [2], [22]. Finally, our findings are limited by the use of a cross-sectional design. Thus, we were unable to determine whether neuroenhancement and illicit drug use in students occurred simultaneously or concurrently.

Notwithstanding these limitations, the present study also has important strengths. The combination of three similar surveys at three different institutions, the use of specific questions regarding the purpose of use (i.e., recreational versus neuroenhancement), and the broad range of substances included in the definition of neuroenhancement are noted strengths of this study. Thus, this study is the first large-scale study of the use of neuroenhancement by Swiss university students and one of the more detailed and comprehensive surveys conducted internationally.

The bioethical debate on neuroenhancement often focuses on reasons for being more liberal with regard to neuroenhancement or defending perspectives on neuroenhancement. Only 15.5% of the respondents in our study were unable to imagine a justification for neuroenhancement. However, other studies reported that most students [42] and also some psychiatrists and general practitioners consider neuroenhancement to be unacceptable [23]. Further research should concentrate on more detailed descriptions of attitudes about neuroenhancement among students and analyze whether students consider pharmaceutical enhancement to be a justifiable behavior, depending on subject characteristics and context. Forty percent of the students in our study accepted the consumption of substances for reasons such as enhancing cognition, alertness, and memory.

Future research should also investigate the prevalence of neuroenhancement in the general population to determine whether only students avail themselves of neuroenhancement strategies or whether employees and apprentices also use prescription drugs or drugs of abuse for the same purposes. We would also like to highlight the importance of future research that carefully compares different studies of neuroenhancing substances because different definitions of the term “neuroenhancement” exist, and the substances and purposes of use vary from study to study. This variation explains to a large extent the different study results with regard to the prevalence of neuroenhancement. New trends in neuroenhancement should also be observed as they develop, and the impact of neuroenhancement on individual situations and behavior should be estimated [43]. If considered necessary by university authorities, for example, health promotion interventions could be developed and implemented based on the empirical data presented in the present study.

## Conclusions

Neuroenhancement with prescription drugs and drugs of abuse is reported by a significant proportion of Swiss university students across academic disciplines. However, in contrast to soft enhancers such as coffee, these substances are rarely used on a daily basis prior to exams. Experience with the use of illicit drugs of abuse was associated with neuroenhancement. Nevertheless, illicit stimulants are primarily used in recreational contexts and rarely for neuroenhancement purposes. Continued observations of the development of cognitive enhancement strategies among students should be made.
